# Trans-hiatal repair for Oesophageal and Junctional perforation: a case series

**DOI:** 10.1186/s12893-020-00702-1

**Published:** 2020-03-02

**Authors:** Adele H. H. Lee, Barry T. S. Kweh, Carla Gillespie, Mary Ann Johnson

**Affiliations:** Upper Gastrointestinal Surgery Unit, St Vincent’s Hospital, Melbourne, Ward 7 East, Upper Gastrointestinal Surgery Unit, Melbourne, Victoria 3065 Australia

**Keywords:** Case reports, Esophageal perforation, Operative time, Laparotomy, Laparoscopy, Thoracotomy

## Abstract

**Background:**

Oesophageal perforation is a life-threatening condition that requires urgent intervention. Surgical repair is recommended within 24 h of onset to minimise mortality risk, traditionally via an open thoracotomy or a laparotomy. Primary oesophageal repair via a laparoscopic trans-hiatal approach has been seldomly reported due to concerns of inadequate eradication of soilage in the mediastinum and pleural space, as well as poor access and an increased operative time in an unwell population.

**Case presentation:**

We report a case series of 3 oesophageal and junctional perforations with varying presentations, demonstrating how the laparoscopic trans-hiatal approach can be used successfully to manage oesophageal perforations.

**Conclusions:**

Laparoscopic trans-hiatal repair is an attractive option for oesophageal and junctional perforations, in haemodynamically stable surgical candidates, in the absence of gross contamination of the thoracic cavity.

## Background

Oesophageal perforation is defined as transmural disruption of the oesophagus [1]. Possible causes include iatrogenic, spontaneous forceful rupture (Boerhaave’s syndrome), malignancy and trauma. Given that symptoms of presentation are often non-specific, clinical diagnosis may be delayed. Classical Computer Tomography (CT) findings include extramural gas locules adjacent to the oesophageal wall, associated with pneumomediastinum or pleural effusion. Early operative intervention within 24 h of onset is recommended given mortality rates up to 40% [2, 3]. We present 3 cases of oesophageal and junctional perforation, managed successfully with laparoscopic trans-hiatal primary repair of the tear.

### Patient selection

**Fig. 1:**
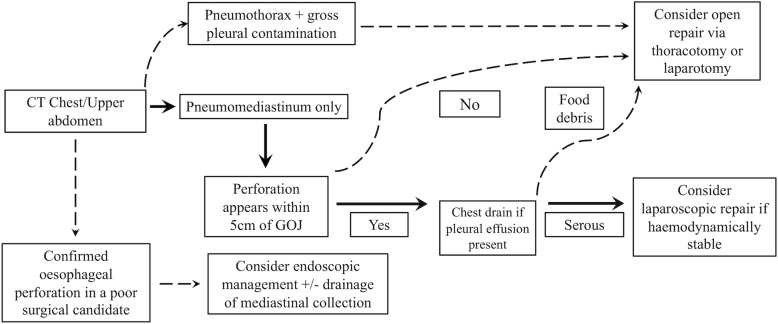
The algorithm we utilised to guide selection of patients for laparoscopic repair of acute oesophageal perforations. A laparoscopic repair is considered in a haemodynamically stable patient if computed tomography chest/upper abdomen suggests that the perforation is contained within the mediastinum, the perforation appears within 5 cm of the GOJ and the pleural effusion, if present, is serous in nature upon drainage. CT = computed tomography. GOJ = gastro-oesophageal junction

We utilised an algorithm to guide patient selection for laparoscopic repair (Fig. 1). When an oesophageal perforation is suspected, a CT chest and upper abdomen with oral contrast is performed to confirm findings. If imaging suggests that the leak is localised within the mediastinum, that the perforation was within 5 cm of the gastro-oesophageal junction (GOJ), and that the pleural effusion if present, is confirmed to be serous in nature upon drainage, a laparoscopic repair is considered if the patient is haemodynamically stable.

### Surgical technique

In all cases, this involved hiatal dissection to access the distal oesophagus. Any fluid present was drained. The tear was exposed and repaired with full thickness interrupted 3–0 Polydioxanone sutures. Intra-operative gastroscopy was performed after control of sepsis, to assess for mucosal closure and for placement of a nasogastric tube for decompression. An omental patch was used to reinforce the suture line. A chest drain was inserted adjacent to the site of the tear in the posterior mediastinum. Antibiotics and fluconazole were continued post-operatively for mediastinitis prophylaxis.

### Case report 1

**Fig. 2:**
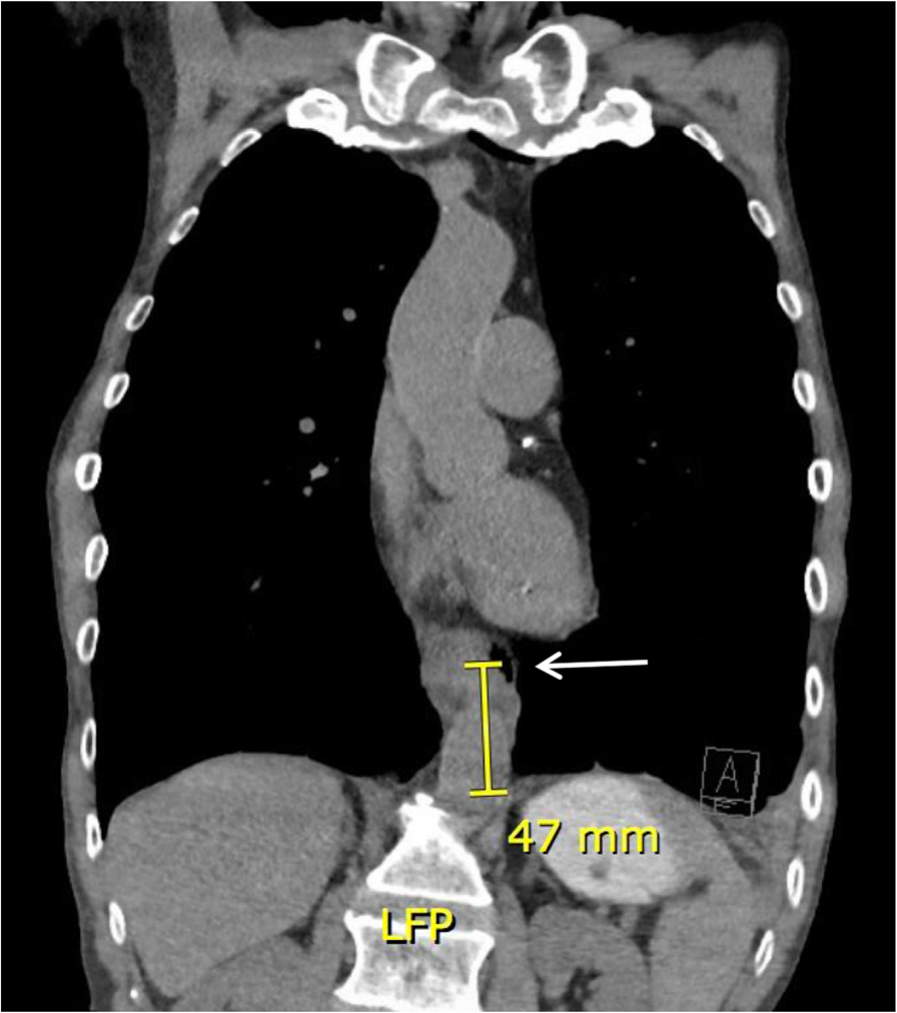
Computed tomography chest demonstrating an extramural gas locule adjacent to the left distal oesophagus about 47 mm from the GOJ (arrow), consistent with perforation. Coronal view. GOJ = gastro-oesophageal junction

A 63-year-old male had presented to a peripheral hospital with throat discomfort, after having ingested a prawn 3 days prior. Past history was significant for alcohol abuse, smoking and diabetes. A gastroscopy performed revealed a food bolus, which had inadvertently been pushed into the stomach during the procedure, causing an oesophageal perforation at 40 to 43 cm from the incisors. At the end of the case, subcutaneous emphysema had developed. He then became confused, febrile and hypotensive, requiring transfer to the intensive care unit (ICU), where he was intubated prior to transfer to our hospital. CT revealed a perforation at the distal oesophagus with pneumomediastinum (Fig. 2). Upon arrival, the patient received an emergency laparoscopic trans-hiatal repair. Intra-operative findings include a full thickness 3 cm tear, 40–43 cm from the incisors, on the anterior wall of the GOJ. A nasojejunal tube was placed intra-operatively for administration of feeds post-operatively. Duration of surgery was 180 min. The patient’s post-operative course was complicated by difficulty in extubation and exacerbation of COPD. He spent 5 days in ICU before being discharged to the ward. The patient was subsequently discharged 11 days post-laparoscopy. Follow-up at 1 month was unremarkable.

### Case report 2

**Fig. 3:**
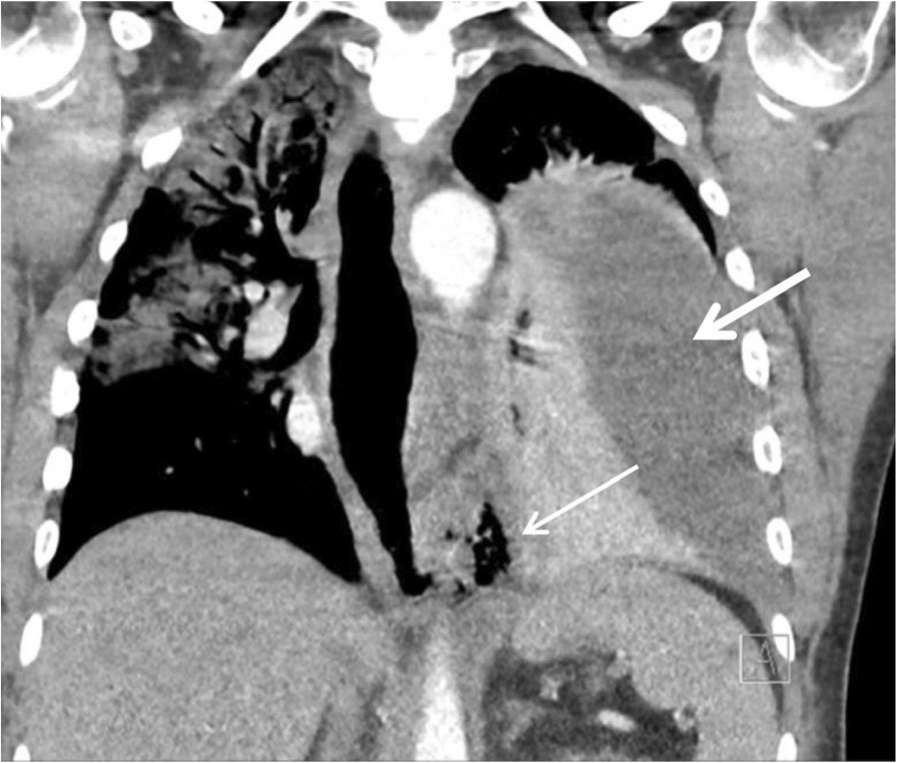
Computed tomography pulmonary angiogram demonstrating a dilated lower oesophagus with a defect at its lateral aspect with gas extending into the left pleural space (thin arrow), consistent with perforation. This is associated with a large left pleural effusion (thick arrow). Coronal view

**Fig. 4:**
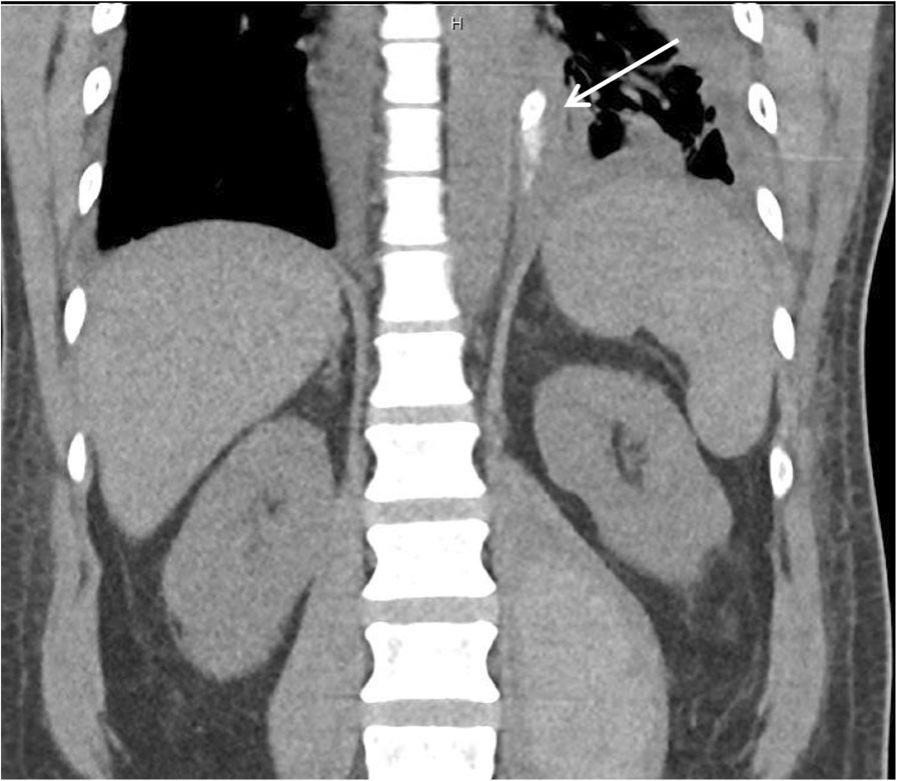
Computed tomography chest/upper abdomen repeated day 7 post-laparoscopy demonstrated extraluminal contrast extending into the left pleural space (arrow). The left pleural effusion has reduced in volume since insertion of intercostal catheter. Coronal view

A 56-year-old male had presented to the emergency department in a state of haemodynamic shock, on the background of polypharmacy overdose and depression. On arrival, he was transferred promptly to the ICU for vasopressor support. He was also put on extracorporeal membrane oxygenation (ECMO) for cardiac and respiratory support, where transoesophageal echocardiography (TOE) was utilised for the 1st 24 h for monitoring. The patient was extubated on day 8 of admission and commenced on full diet. On day 9, the patient experienced chest pain after eating, associated with tachycardia and a raised white cell count (WCC) of 30 × 10^9^/L and C-reactive protein (CRP) of 303 mg/L. CT pulmonary angiography (CTPA) revealed findings consistent with oesophageal perforation, with gas extending into the pleural space, associated with a large left pleural effusion (Fig. 3). A chest tube inserted to drain the effusion returned with serous fluid, which excluded gross contamination of the pleural space. The patient was subsequently planned for an emergency laparoscopic trans-hiatal repair. He was haemodynamically stable and off vasopressor support prior to surgery, with a blood pressure of 160/80. Gastroscopy performed on the table demonstrated circumferential ‘grey’ mucosa 25 to 40 cm from the incisors (Gurvits Syndrome). Duration of surgery was 300 min. This was prolonged due to the debridement of necrotic tissue as well as formation of a feeding jejunostomy for administration of feeds post-operatively. Location of the tear in the mediastinum also resulted in a longer time spent on hiatal dissection. The patient’s post-operative course was complicated by acute kidney injury and ischaemic hepatitis secondary to septic shock from hospital acquired pneumonia. Due to ongoing high drain outputs, a CT with oral contrast was performed day 7 post-laparoscopy, which revealed extraluminal contrast into the left pleural space consistent with ongoing leak (Fig. 4). This was treated conservatively and had resolved on repeat imaging 7 days later. Additionally, a gastroscopy performed to investigate ongoing dysphagia identified a stricture at 25 to 40 cm from the incisors, likely secondary to ischemia and the oesophageal leak. This was dilated with Savary Gillard dilators. The patient spent 15 days in ICU before being transferred to the ward. He was discharged 49 days post-laparoscopy. For the past year, the patient has been undergoing monthly dilatations as management of the persistent oesophageal stricture.

### Case report 3

**Fig. 5:**
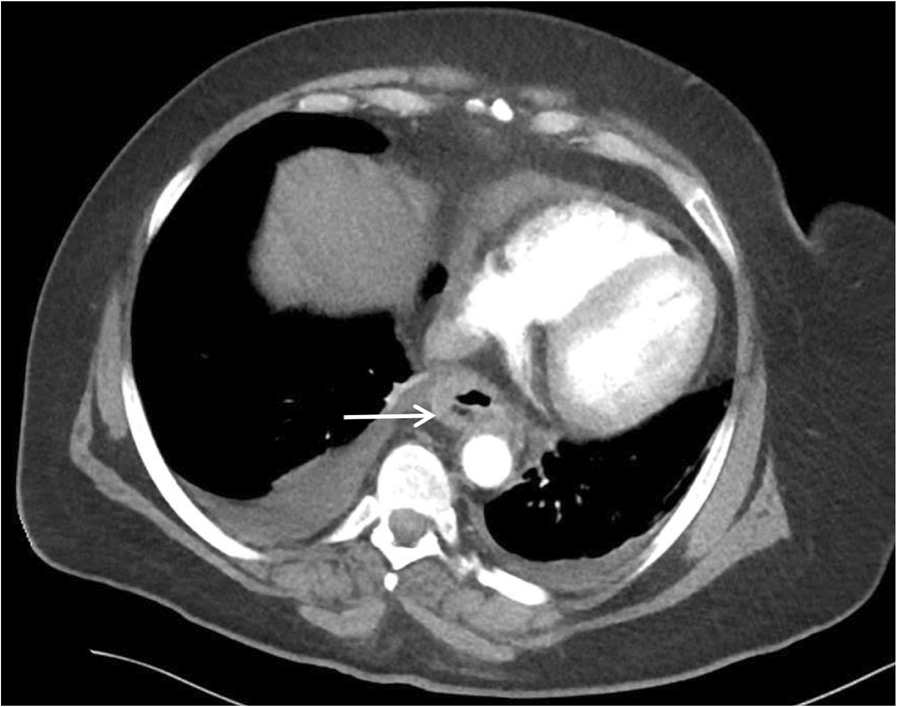
Computed tomography chest revealed extra-mural fat stranding and gas locules to the right lateral aspect of the gastro-oesophageal junction (arrow). Axial view

A 51-year-old female was initially admitted for cauda equina syndrome secondary to epidural lipomatosis, for which she received an emergency laminectomy. Day 1 post-laminectomy, the patient experienced symptoms of sore throat, dysphagia and chest pain. Examination was unremarkable. WCC was 10.1 × 10^9^/L and CRP was 581 mg/L. Initial CT revealed fluid along a distended thick-walled oesophagus concerning for oesophagitis. Persistence of symptoms and rising inflammatory markers despite antibiotics led to a repeat CT 6 days later, which then revealed extramural gas locules with associated fat stranding adjacent to the GOJ, consistent with perforation (Fig. 5). She underwent an emergency laparoscopic trans-hiatal repair. Intra-operative findings included a 2 cm mucosal tear at the superior lesser curve extending to the GOJ. A nasojejunal tube was placed intra-operatively for administration of feeds post-operatively. Duration of surgery was 180 min. Her post-operative course was complicated by a pericardial effusion, which was treated with a pericardial window. She was discharged 34 days post-laparoscopy. Follow-up at 4 months was unremarkable. Gastroscopy revealed a well-healed gastric ulcer at the cardia.

## Discussion and conclusions

Oesophageal perforation can be traumatic or spontaneous. Traumatic perforation may be iatrogenic as in our cases, caused by intra-oesophageal instrumentation or inflammation. Perforation most often occurs at the left posterolateral wall of the distal oesophagus, 2 to 3 cm from the GOJ. It carries a higher mortality rate, with complications such as mediastinitis, pleuritis and sepsis. This is further exacerbated by misdiagnosis of similar presenting pathologies including myocardial infarction or a perforated ulcer, leading to delayed treatment [4]. A 12-year national study of oesophageal perforations in England demonstrated a 30-day and 90-day mortality rate of 30 and 39% respectively. However advancements in management and centralisation of care has led to an overall decrease in mortality over the years [5].

The aim of treatment is sepsis control and closure of the oesophageal tear. A variety of management options exist, such as conservative measures, radiological interventions, endoscopic management, vacuum therapy and surgery. Management can be guided with the Pittsburgh perforation severity score (PPSS). Surgery is recommended in those with intermediate and high PPSS, which is highly suggestive of mediastinitis, given their high rate of failure of conservative management (29 and 71% respectively) [6]. It is recommended within 24 h as after this, sutures are unlikely to hold due to tissue necrosis and oedema. However, there are conflicting reports on whether surgery after 24 h increases mortality rates. Therefore, time frame should not be the sole factor guiding management [7, 8]. The gold standard approach to primary repair is via open thoracotomy or laparotomy. These approaches are favoured due to thoughts of optimal control of sepsis and draining the area of soilage [4].

Minimally invasive techniques have been demonstrated to have favoured outcomes in resections for oesophageal cancer, with significant reductions in post-operative pulmonary complications, but there is little evidence available for emergency repair for oesophageal perforation [2]. Benefits other than minimised morbidity due to smaller incisions also include lower risks of wound infection and wider exposure. Such techniques should only be utilised in haemodynamically stable surgical candidates. Septic patients should be resuscitated and optimised with antibiotics prior to surgery [9]. It should generally be utilised where contamination is localised within the mediastinum, as removal of contaminants may not be sufficient otherwise [10]. In the presence of a mediastinal abscess, percutaneous drainage can be utilised as an adjunct, having demonstrated high success rates without the need for re-intervention in up to 96% of patients [11, 12].

Thoracoscopy has been shown to be effective in controlling sepsis [2, 8, 13]. A cohort study by Elliot et al. of patients presenting with Boerhaave’s syndrome over 6 years, managed with thoracoscopic debridement and primary repair, achieved a favourable mortality rate of 10% [2]. Similarly, Nakano et al. demonstrated that thoracoscopic primary repair with mediastinal drainage had acceptable duration of surgery and post-operative outcomes [8]. However, this approach does not allow for reinforcement of the suture line with an omental patch - this would require re-positioning and abdominal access [14]. Its use is limited to small tears, about 2–3 cm in diameter [10].

Endoscopic management of oesophageal perforation also offers an option with reduced morbidity and cost, associated with equally effective results. A systematic review by Kamarajah et al. has reported technical and clinical success rates of 96 and 87% with esophageal stents [15]. Even though this technique prevents ongoing contamination, it cannot remove mediastinal soilage that is already present. Hence, in surgical candidates who can tolerate a definitive procedure, definitive repair is considered to control sepsis and prevent deterioration. Stent migration, compromising healing, is another concern, and poses a high risk when inserted in a perforated benign oesophagus compared to the malignant cohort, with rates of up to 12% [16]. For this reason also, perforations in the distal oesophagus or close to the GOJ are not managed in this way. Other relative contraindications include long segment perforations (> 6 cm), a patient requiring immediate thoracotomy for an associated injury, oesophageal injuries in the cervical region, as well as near complete dehiscence or evidence of necrosis in the oesophageal wall [17]. In all 3 of our cases where perforations involving the distal oesophagus were associated with localised mediastinal contamination in surgical candidates, endoscopic management was not the preferred management option. Endoscopic therapy can be considered if a patient is a poor surgical candidate, or in the setting of an iatrogenic perforation noted during endoscopy, which is small (≤ 6 mm) with healthy edges.

In contrast, laparoscopic repair for oesophageal perforation is less well described in the literature. Published case reports have reported successful management of oesophageal perforation via laparoscopy, with benefits that outweigh that of thoracoscopy or endoscopy [4, 9, 10, 18–20].

The laparoscopic approach allows greater exposure to be obtained to identify the perforation site, as compared to laparotomy or a transthoracic approach. This can be attributed to mediastinal dissection being performed under direct vision and versatility obtained with the 30° laparoscope and long instruments [18]. As opposed to the transthoracic approach, it allows for the use of the greater omentum to reinforce the primary suture repair, which is recommended in cases at higher risk of leak, such as in sepsis or when diagnosis is delayed, and where the perforation site is larger than 3 cm [19]. In cases where suturing is considered unsafe, a fundic patch with a posterior fundoplication has been used to cover the tear to protect the mucosal breach from physiological reflux [20]. The laparoscopic approach also allows for drain placement in the posterior mediastinum and for assessment of the intra-abdominal extent of the injury [9, 10]. It is noted that pleural effusions cannot be washed out with this approach. Despite this, the literature has not reported any cases which required pleural decortication following this approach. Two cases have required further thoracoscopic drainage and a pleural tap due to re-presentations with CT findings of a left-sided empyema and pleural effusion respectively. After which, symptoms resolved with targeted antibiotic and antifungal therapy based on pleural fluid analysis with no further re-interventions required [19, 21]. Hence, we do suggest confirming that the pleural effusion if present pre-operatively is serous in nature prior to consideration for laparoscopic repair.

Pre-operative localisation of the tear is recommended prior to laparoscopy as this technique may not reach the upper end of tears that extend too far into the thorax, or control sepsis where gross contamination of the thoracic cavity has occurred [20]. Computed tomography (CT) plays an important role in guiding management particularly in assessing the location, size, and extent of contamination. The sensitivity of CT in diagnosis of a perforation ranges from 50 to 100% [22–24]. Contained perforations or mucosal tears do prove to be more of a challenge to diagnose, which contribute to decreased sensitivity. However, false negatives may be avoided by optimising CT protocols, as well as the use of oral contrast. This has to be balanced with the risk of aspiration and development of pulmonary complications [25]. Overall, CT is a useful tool in assessment and plays an important role in selecting patients for laparoscopic repair.

Despite the laparoscopic approach being described many years ago, it has been poorly adopted due to the paucity of strong evidence advocating its use. There are observational studies available demonstrating non-inferiority between laparoscopic and open oesophagectomy for oesophageal cancer [26]. The lack of observational studies or trials involving acute oesophageal perforations is due to the low incidence of cases encountered to demonstrate non-inferiority for laparoscopic repair. We propose that the next step to establishing the value for this approach would be conducting a multi-site feasibility study of patients who meet the criteria outlined by our algorithm, measuring primary outcomes including post-operative morbidity and mortality rates, length of hospital stay, readmission or re-intervention rate, as well as the cost burden, in order to demonstrate the safety as well as the cost-effectiveness to this approach.

Laparoscopic trans-hiatal repair is an attractive option for oesophageal and junctional perforations, in haemodynamically stable surgical candidates, in the absence of gross contamination of the thoracic cavity. It should be considered early, within 24 h of onset of symptoms, after perforation has been confirmed.

## Data Availability

Not applicable.

## Abbreviations

CRP: C-reactive protein
CT: Computer Tomography
CTPA: CT pulmonary angiography
ECMO: Extracorporeal membrane oxygenation
ICU: Intensive care unit
PPSS: Pittsburgh perforation severity score
TOE: Transoesophageal echocardiography
WCC: White cell count
